# Medical Properties and Uses of the Cimicifuga Racemosa

**Published:** 1851-12

**Authors:** E. M. Brundige


### MATERIA MEDICA AND THERAPEUTICS.

*Medical Properties and Uses of the Cimicifuga Racemosa.* By E. M. BRUNDIGE, M. D.—As an article of the Materia Medica, this plant is of comparatively recent introduction, and its medical properties seem hitherto to have been imperfectly investigated. Mild tonic virtues have been ascribed to it as well as the property of exciting the secretions of the skin, kidneys, and bronchial tubes. Emetic and cathartic operations are likewise said to have been produced by it, but I have never observed these effects; perhaps, because my doses have been too small, or the preparation chiefly used has been the alcoholic, and not the proof spirit tincture. Alcohol takes up from this root very little coloring matter, and perhaps it rejects something upon which its emetic properties depend. But of its narcotic principle it is a perfect solvent. The therapeutical effects that have been ascribed to this, in my opinion, would receive a ready solution on the supposition that it acts only as a narcotic. That it possesses this property to a considerable degree, can scarcely be doubted. It is a stimulant narcotic, in over doses producing vertigo even to falling, from which the patient soon recovers. When any tendency to cerebral plethora exists, it is one of the worst remedies that can be used, never failing to increase headache and vertigo. In the diseases depending upon innervation, it is one of the best remedies. It is a very prompt and efficient remedy in many cases of atonic and irritable dyspepsia, in doses of grs. x of the powder three times daily. If it has proved decidedly useful in pulmonary cases, as has been asserted, it has probably been when the lung affection depended on irritation in the digestive organs. Its curative effects in subacute and chronic rheumatism are unquestionable. In the treatment of this complaint, as well as in neuralgia, it is often advantageous to combine with it as much colchicum as may be required to keep up sufficient action of the bowels. The tincture of hyoscyamus has been added with happy effect in cases of rheumatism, attended by unusual pain and irritation. A liberal application of the tincture to the surface, both in rheumatism and neuralgia, has been found very useful. Internally, the powder should be administered in grs. xx doses, three or four times a day. The quantity may be increased if necessary. In chorea, I have not seen it tried, but should expect it to exert a controlling influence. Parturient properties have been ascribed to it, and not without cause. As a means of increasing uterine action, it is far less energetic than ergot, but often a better remedy, since the energy with which ergot acts is frequently an objection to its use. In cases requiring but moderate aid, a teaspoonful of the tincture, given for two or three successive half hours, will, in a short time, excite steady and efficient action, which usually lasts till delivery. As a means of detecting false labor pains, it is very valuable, as it soon arrests them, restoring to the patient comfortable and healthy feelings. The frequent vomiting often so annoying in parturition is immediately relieved by it, while the uterine action is increased.

The foregoing are, I believe, about all the purposes for which this article has been used, and are of themselves of sufficient importance to establish its character as a remedial agent. I am now about to present it in a new and far more interesting light, and to state facts to show that, in the treatment of one disease at least, it posesses most extraordinary powers.

I have been accustomed to see this remedy applied to the inflammatory diseases of the eye of the horse, as well as to inflammations in other parts, from early youth, with the most complete success, the inflammation subsiding in from twenty-four to forty-eight hours. In these cases, the decoction was used, the parts being kept constantly wet with it. Dr. Close, of Portchester, with whom I studied, first applied it to the human eye, and to him I am indebted for valuable statistics.

Ophthalmia, in its various stages and degrees, but especially in its recent and acute form, is the disease over which the cimicifuga has been found to exert an influence so decisive and so prompt, as to establish for it a claim of superiority over every other mode of treatment. By the application of two or three drachms of the tincture to the region of the eye, the disease has been overcome in twenty-four hours. Cures just as prompt and complete have often been made, not rarely and in a few equivocal cases, but constantly, as often as the disease has occurred; and in its most aggravated forms. This has been done unaided by any other means, not even by a purgative, but solely by the application of this simple and apparently trivial remedy. To secure a result so desirable, a saturated alcoholic tincture of the dried root is indispensable. A weaker tincture will not succeed unless the quantity is considerably increased, and then the stimulus of the alcohol in sensitive persons is sometimes so great as to increase the disease, and thus prevent a cure. The proper quantity to be applied, when the inflammation is confined to one eye, is a drachm, and twice the quantity when both eyes are affected. It should be applied slowly and cautiously with a pencil brush, the eyelids being closed so as to prevent, as far as possible, the liquid penetrating between them. Should this occasionally happen, the temporary smarting will not be followed by any unfavorable effect. The brush, when first charged, should be applied to the edge of the hair, at the greatest distance from the eye, passed entirely around it, approaching nearer as the fluid becomes exhausted, and finishing over the eyelids. This course should be repeated until the liquid is all applied, suspending the process now and then to afford the skin time to dry. The application should never be hurried, nor the surface to which it is applied too limited, but should comprise the forehead, temples, superior maxillary region, sides of the nose and eyelids. Its application to so extended a surface is necessary, that all the nerves around the eye may be brought under its influence, and through their action on the blood-vessels, the circulation of the inflamed organ be restored to its normal state. In recent and not very severe cases, one application is sufficient, but in more aggravated cases, it should be repeated after a lapse of two or three hours, and occasionally may be required the second day. In chronic cases the application must be repeated for several successive days, although cases of five weeks' standing have yielded to a single application. In very old and especially chronic cases, the power of the remedy has been greatly enhanced by being used in conjunction with the ointment of iodide of potassium, the tincture being applied first, and the ointment immediately after. In cases attended with unusual pain and irritability, the addition of sulph. morph. grs. iv. or v. to the ℥j of the tincture will be found very useful. When the skin about the eyelids is very red and sensitive, with a tendency to excoriation, so that from the stimulus of the alcohol the application can scarcely be borne, dissolving tannin grs. vii. to xii. in the tincture will immediately obviate the difficulty. The class of cases in which this remedy shows the most marked effect, is that in which the disease is of an acute form attended by great pain and intolerance of light. The first case to which this treatment was applied, was that of a little boy, who was lying in a darkened room with his eyes firmly closed, and unable to open them. On a forcible inspection, the sclerotic coat was seen gleaming through a flood of tears of a fiery red color. Extreme reluctance to submit so young a child to the usual treatment, induced the practitioner to try the tincture of cimicifuga, having previously witnessed its curative influences in some other forms of local inflammation. It was accordingly applied in the manner above described, and repeated in the afternoon. On calling the following morning, the mother of the little boy was leading him about the yard, his eye entirely cured. Results as striking as this have followed every case when a proper application has been made. I have never yet failed in a single case. Dr. Close, in a practice of five years, gives only one case in which the disease resisted its influence, and that yielded to the remedy after the usual depletory treatment, and in the village where he resides numerous cases occur.

In the purulent form of ophthalmia, but one opportunity has occurred in which I have applied it. Four cases occurred in a family, contracted from a colored girl who came among them, partially cured, from Bellevue Hospital. Three of these yielded, in about a week, to the daily application of the tincture. The fourth was very obstinate, confining the patient to a dark room for three or four weeks, but gave way at last to blisters to the back of the neck, and quinine internally. During the whole of this period the pain and suffering were greatly relieved by the external application of the tincture, applied at any time when the pain became more aggravated.

Should any of my medical brethren be induced to try this mode of treatment, I must insist upon the observation of the precautions above pointed out, both as to the strength of the tincture and the mode of applying it, and I would especially caution them against leaving the application to the patient or his friend. A properly prepared tincture must be used, the root having been gathered within six months.

A case of neuralgia successfully treated by cimicifuga, will close my observations on the use of this article. A young lady had been suffering for more than two years with this disease in the third branch of the fifth pair, for which, early in the disease, a sound molar tooth had been extracted, because the pain always concentrated there. The pain continued to return in daily paroxysms. The usual treatment was resorted to with but slight relief. The last article used was the tincture of the cimicifuga, in doses of a drachm, three or four times a day internally, the same being applied externally, about the same number of times. In three weeks the pain had entirely ceased. Thirteen months afterwards, it returned on the opposite side of the face, and was effectually removed in a few days by the same treatment, no return of the disease having occurred for the last three years.—*N. Y. Medical Gazette.*

